# Supplementary material for: Modeling glioblastoma heterogeneity as a dynamic network of cell states
Source: Mol Syst Biol. 2021 Sep 16;17(9):e10105. doi: 10.15252/msb.202010105 (PMC8444284; doi:10.15252/msb.202010105)
Supplement: Supplementary file 6 — Source Data for Figure 5 [file MSB-17-e10105-s004.zip › Figure5A_sourcedata/GSEA_3071/nosigdb_stateVI.GseaPreranked.1622795169056/NB_KI_CLUSTER12.html]

Details for gene set NB\_KI\_CLUSTER12[GSEA]

|  || Dataset | state63071 |
| Phenotype | NoPhenotypeAvailable |
| Upregulated in class | na\_pos |
| GeneSet | NB\_KI\_CLUSTER12 |
| Enrichment Score (ES) | 0.3876711 |
| Normalized Enrichment Score (NES) | 1.9217018 |
| Nominal p-value | 0.009049774 |
| FDR q-value | 0.008448854 |
| FWER p-Value | 0.079 |
Table: GSEA Results Summary

  

Fig 1: Enrichment plot: NB\_KI\_CLUSTER12      
 Profile of the Running ES Score & Positions of GeneSet Members on the Rank Ordered List

  

| PROBE | GENE SYMBOL | GENE\_TITLE | RANK IN GENE LIST | RANK METRIC SCORE | RUNNING ES | CORE ENRICHMENT || 1 | RGCC |  |  | 17 | 1.065 | 0.0664 | Yes |
| 2 | COTL1 |  |  | 60 | 0.851 | 0.1116 | Yes |
| 3 | LPXN |  |  | 85 | 0.772 | 0.1564 | Yes |
| 4 | TRAC |  |  | 87 | 0.768 | 0.2074 | Yes |
| 5 | FXYD5 |  |  | 107 | 0.732 | 0.2510 | Yes |
| 6 | CLEC2B |  |  | 132 | 0.688 | 0.2902 | Yes |
| 7 | EZR |  |  | 148 | 0.667 | 0.3306 | Yes |
| 8 | BIRC3 |  |  | 191 | 0.608 | 0.3596 | Yes |
| 9 | ZFP36L2 |  |  | 284 | 0.547 | 0.3706 | Yes |
| 10 | TNFAIP3 |  |  | 445 | 0.473 | 0.3579 | Yes |
| 11 | CD55 |  |  | 452 | 0.471 | 0.3877 | Yes |
| 12 | ABCC4 |  |  | 737 | 0.389 | 0.3350 | No |
| 13 | MAPKAPK3 |  |  | 785 | 0.379 | 0.3473 | No |
| 14 | TRABD2A |  |  | 984 | 0.348 | 0.3157 | No |
| 15 | CRYBG1 |  |  | 1038 | 0.339 | 0.3237 | No |
| 16 | ICAM3 |  |  | 1177 | 0.319 | 0.3068 | No |
| 17 | ABRACL |  |  | 1354 | 0.297 | 0.2779 | No |
| 18 | RHOG |  |  | 1355 | 0.297 | 0.2977 | No |
| 19 | ISG20 |  |  | 1756 | 0.255 | 0.2039 | No |
| 20 | CREM |  |  | 2019 | -0.282 | 0.1502 | No |
| 21 | IQGAP2 |  |  | 2166 | -0.308 | 0.1303 | No |
| 22 | STK17A |  |  | 2336 | -0.340 | 0.1062 | No |
| 23 | PREX1 |  |  | 2727 | -0.432 | 0.0271 | No |
| 24 | PDE4B |  |  | 2948 | -0.513 | 0.0004 | No |
| 25 | ITGA4 |  |  | 3132 | -0.610 | -0.0096 | No |
| 26 | CXCR4 |  |  | 3302 | -0.759 | -0.0057 | No |
| 27 | CEMIP2 |  |  | 3573 | -1.475 | 0.0180 | No |
Table: GSEA details [plain text format]

  

Fig 2: NB\_KI\_CLUSTER12: Random ES distribution      
 Gene set null distribution of ES for **NB\_KI\_CLUSTER12**
